# Mulberry (*Morus alba*) Twig and Leaf Extracts Ameliorate Obesity-Related Metabolic Disorders via Gut Microbiota Modulation in High-Fat Diet-Fed Mice

**DOI:** 10.3390/ani15121768

**Published:** 2025-06-15

**Authors:** Wei Qian, Jinyan Han, Xiang Shi, Xiaoqing Qin, Feng Jiao, Minjuan Zhang, Lijun Bao, Chao Su

**Affiliations:** College of Animal Science and Technology, Northwest A&F University, Yangling 712100, China; qianvii@163.com (W.Q.); hanjinyan0805@163.com (J.H.); shix0035@163.com (X.S.); qinxq3524@163.com (X.Q.); fjiao@nwsuaf.edu.cn (F.J.); mjzhang1008@nwsuaf.edu.cn (M.Z.)

**Keywords:** mulberry extract, glycolipid metabolism, gut microbiology, obesity

## Abstract

Mulberry (*Morus alba*) twigs and leaves, rich in flavonoids, polyphenols, polysaccharides, and alkaloids with multi-target regulatory properties on glucose/lipid metabolism, were evaluated for their anti-obesity effects using methanol-extracted twigs (MTE) and aqueous-extracted leaves (MLE) in high-fat diet (HFD)-induced obese mice. Both extracts significantly ameliorated obesity-related metabolic dysregulation, as evidenced by attenuated body weight gain, visceral fat accumulation, serum lipid profiles, homeostatic model assessment of insulin resistance (HOMA-IR), and hepatic inflammation compared to HFD controls (*p* < 0.05). Concurrently, MTE and MLE enhanced systemic antioxidant capacity and elevated high-density lipoprotein cholesterol (HDL-C) levels. Notably, high-dose MTE (MTEH, 1000 mg/kg) markedly reduced perirenal adiposity while increasing brown adipose tissue mass (*p* < 0.05). Mechanistic investigations revealed that MTEH reshaped gut microbiota composition by suppressing *Firmicutes* and *Enterococcus*, while enriching beneficial *Faecalibaculum* and *Bifidobacterium* spp. (*p* < 0.05). Furthermore, cecal short-chain fatty acid (SCFA) profiling demonstrated MTEH and MLEH-mediated metabolic reprogramming, characterized by increased propionic acid and decreased butyric acid, suggesting microbiota-dependent modulation of host energy metabolism. These findings collectively highlight the potential of mulberry extracts as multi-targeted nutraceuticals for obesity intervention via gut microbiota–SCFA axis regulation.

## Simple Summary

The present study investigated the anti-obesity effects of methanol-extracted mulberry twigs (MTE) and aqueous-extracted mulberry leaves (MLE) in high-fat diet (HFD)-induced obese mice. Phytochemical analysis revealed that both extracts contained bioactive flavonoids, polyphenols, polysaccharides, and alkaloids with known regulatory effects on glucose/lipid metabolism. It was shown that mulberry twigs and mulberry leaf extracts alleviated high-fat diet-induced obesity through gut microbiota remodeling and SCFA metabolic reprogramming, and that mulberry twigs and mulberry leaf extracts enriched E. faecalis and Bifidobacterium abundance while suppressing obesity-associated genera, significantly increased cecum propionic acid content, and significantly reduced butyric acid content in obese mice, providing a sustainable strategy for value-adding agricultural by-products.

## 1. Introduction

Excessive lipid deposition in livestock constitutes a major metabolic inefficiency where dietary energy is preferentially channeled into fat storage rather than productive growth. This metabolic dysregulation significantly reduces feed conversion efficiency, elevates production costs, and ultimately diminishes livestock performance [1]. The condition is particularly problematic in modern intensive farming systems where optimal nutrient utilization is crucial for economic viability.

In poultry production, lipid accumulation establishes a bidirectional relationship with gut microbial dysbiosis [2]. High-fat diets consistently disrupt intestinal microbiota homeostasis, typically manifesting as an elevated Firmicutes/Bacteroidetes ratio, depletion of beneficial bacterial populations (e.g., Lactobacillus and Bifidobacterium), and proliferation of potentially pathogenic species [3,4]. This dysbiotic state further aggravates lipid deposition through multiple mechanisms: compromising intestinal barrier function, diminishing short-chain fatty acid (SCFA) production (particularly of butyrate), and disrupting bile acid metabolism [5,6,7]. Concurrently, the metabolic disturbances from excessive lipid deposition create a self-perpetuating cycle that exacerbates intestinal ecological imbalances [8,9,10].

The intricate host–microbiome interaction not only impairs nutrient absorption efficiency but also exacerbates systemic metabolic disorders through the gut–liver axis [11,12]. Species-specific manifestations include the following: in avian species, microbiota dysbiosis triggers TLR4/NF-κB pathway activation, leading to intestinal inflammation and pronounced hepatic lipid accumulation [13,14]; modifications in bile acid metabolism substantially impact lipid digestion and absorption efficiency [15]; and in ruminants, alterations in rumen microbial composition significantly alter volatile fatty acid profiles, thereby regulating lipogenic processes [16,17]. These findings underscore the critical need for targeted interventions to break this metabolic vicious cycle.

Phytogenic compounds have emerged as promising candidates for metabolic regulation, with mulberry (*Morus alba*) being particularly noteworthy [18,19,20]. This medicinal plant contains a diverse array of bioactive components including polyphenols [21], flavonoids [22], anthocyanins [23], alkaloids [24], and polysaccharides [25], which collectively contribute to its multifaceted pharmacological effects. Numerous studies have documented mulberry’s therapeutic potential in metabolic regulation: mulberry leaf aqueous extract (MLE) significantly attenuates oxidative stress and improves lipid profiles in obese models by modulating FASN expression and enhancing hepatic antioxidant enzymes [26,27]; black mulberry extract demonstrates potent anti-lipogenic effects through AMPK/mTOR pathway regulation [28,29]; while mulberry twig alkaloids (SZ-As) show remarkable efficacy in ameliorating diabetic complications through gut microbiota modulation [30,31].

Mulberry leaf extract (MLE) offers distinct advantages in terms of production cost-efficiency and simplified processing procedures. In contrast, mulberry twig methanol extract (MTE) demonstrates superior efficacy in recovering bioactive compounds, particularly through its enhanced capacity to capture both polar phytochemicals (e.g., polyphenols) and moderately non-polar constituents (e.g., specific alkaloid classes), as evidenced by comparative studies [32,33,34]. Processed extracts present significant pharmacological advantages over crude plant materials, including the improved bioavailability and mitigation of practical challenges associated with direct plant administration. Notably, these refined formulations effectively address palatability concerns while eliminating anti-nutritional factors typically encountered in crude mulberry bran preparations [35,36,37]. Thus, we investigated the potential of mulberry branch and leaf extracts as functional feed additives by evaluating their therapeutic effects in high-fat diet (HFD)-fed mice.

## 2. Materials and Methods

### 2.1. Extract Preparation

Mulberry twigs were obtained from the mulberry field of Zhouzhi Erqu Experimental Station of Northwest Agriculture and Forestry University (Yangling District, Shaanxi Province, China). Mulberry twigs and leaves were extracted according to the extraction procedure of a material–liquid ratio of 1:20, an extraction time of 2 h, a methanol concentration of 60%, and an extraction temperature of 80 °C. The extracts were batch extracted by Xi’an Bo’ao Xintian Botanical Development Co., Ltd. (Xi’an, China) according to the optimized protocol of our laboratory. The active ingredients of MLE include 10.8% alkaloids, 18.57% flavonoids, 11.96% terpenoids, 9.71% phenolic acids, 8.95% lipids, 6.18% lignin and coumarin, 2.97% organic acids, 2.64% nucleotides and their derivatives, 1.52% quinones, 0.23% steroids, 0.17% tannins, etc. MLE includes alkaloidal constituents (10.80%), flavonoid derivatives (19.37%), terpenoid compounds (11.60%), phenolic acids (10.42%), lipid components (8.82%), lignans and coumarin derivatives (6.15%), organic acids (2.93%), nucleoside-related metabolites (2.39%), quinone-containing compounds (1.69%), tannins (0.29%), and steroidal components (0.22%). No methanol residues were detected in MTE and the residue levels were well below the stringent safety threshold of 0.3% set by the International Pharmacopoeia standard (ICH Q3C Guidelines) [38].

### 2.2. Materials and Instruments

The maintenance diet for experimental rats (JY-B30630040 3.79 kcal/g) was purchased from High Education and Research Technology Co., Ltd. (Beijing, China); the high-fat diet (H10060, 5.24 kcal/g) was purchased from Chongqing Tengxin Biotechnology Company Limited (Chongqing, China). The maintenance diet ‘s energy ratio includes 21.5% protein, 11.1% fat, and 67.4% carbohydrates. In contrast, the high-fat diet’s energy ratio includes 20% protein, 60% fat, and 20% carbohydrates; and the blood glucose meter and blood glucose meter test strips were purchased from Shanghai Kandeler Pharmaceuticals Co. (Shanghai, China).

### 2.3. Animal Handling and Grouping

**Figure 1:**
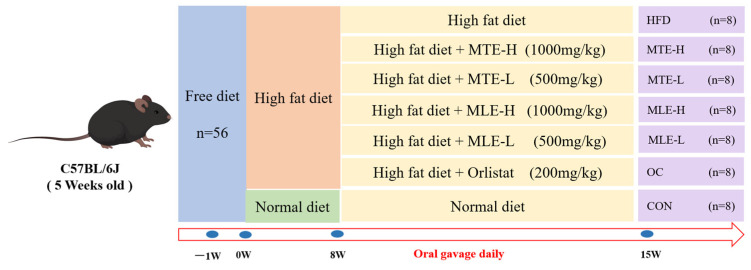
Design of the experiment. Mulberry twig extract high dose, 1000 mg/kg group (MTEH); mulberry twig extract low dose, 500 mg/kg group (MTEL); mulberry leaf extract high dose, 1000 mg/kg group (MLEH); mulberry leaf extract high dose, 500 mg/kg group (MLEL); orlistat 200 mg/kg group (OC); blank control group (CON, normal diet + equal amount of distilled water); and high-fat diet feeding group (HFD, high-fat diet + equal amount of distilled water).

The entire process of animal experiments was conducted in strict accordance with the National Research Council Guidelines for the Care and Use of Laboratory Animals and was approved by the Animal Ethics Committee of Northwest Agriculture and Forestry University (Approval No. XN2024-0102, 2024). These mice were housed in the Laboratory Animal Center of Northwest Agriculture and Forestry University at a temperature of 23 ± 1 °C with a 12 h light/dark cycle. After one week of acclimatization, 56 mice were randomly divided into 7 groups according to their body weight (20 ± 1 g): high-fat diet + distilled water (HFD), high-fat diet + 200 mg/kg orlistat (OC), high-fat diet + 1000 mg/kg MTE (MTE-H), high-fat diet + 500 mg/kg MTE (MTE-L), high-fat diet + 1000 mg/kg MLE (MLE-H), high-fat diet + 500 mg/kg MLE (MLE -L).CON (basal diet + equal amount of distilled water), and HFD groups (HFD + equal amount of distilled water); each group of mice was divided into 5 cages with 5 mice per cage, and the MTE used in this study was determined based on preliminary and related experiments. The experimental group was fed the HFD for 8 consecutive weeks to establish an obesity model, and the blank control group was fed the basal diet throughout the experimental period. The experimental design is shown in Figure 1.

During the study period, daily gavage was administered at 9:00 a.m. Body weight (BW) and 6 h-fasted blood glucose (FBG) were measured weekly. During the final treatment week, all mice underwent an oral glucose tolerance test (OGTT). Subsequently, mice were anesthetized, and fecal samples, blood, and tissues were collected.

### 2.4. Biochemical Analysis

Mouse blood samples were collected, allowed to stand, and centrifuged. Aspartate aminotransferase (AST, MAK055, Merck, Darmstadt, Germany), alanine aminotransferase (ALT, MAK052, Merck), total cholesterol (TC, BC1985, Solarbio, Beijing, China), and low-density lipoprotein cholesterol (LDL-C, 60736ES, Yesen, Shanghai, China), high-density lipoprotein cholesterol (HDL-C, 60736ES, Yesen, Shanghai), and triglycerides (TG, S0219S, Beyotime, Shanghai, China) were measured with an autoanalyzer (BS-1000, Myriad, Shenzhen, China) according to the manufacturer’s guidelines. Homeostasis model assessment of insulin resistance (HOMA-IR) was determined using the following equation: HOMA-IR = FBG (mmol/L) × FINS (μU/mL)/22.5. Hepatic inflammatory cytokines including Leptin (LEP), Insulin (INS), adiponectin (APN), levels of interleukin-6 (IL-6), interleukin-8 (IL-8), interleukin-17 (IL-17), tumor necrosis factor-α (TNF-α), malondialdehyde (MDA), glutathione peroxidase (GSH-Px), superoxide dismutase (T-SOD), and total antioxidant capacity (T-AOC) in the liver were determined using an enzyme-linked immunosorbent assay (ELISA) kit (Shanghai Vankevi Bio-Technology Co., Shanghai, China)

### 2.5. Histological Analysis of Liver

Liver tissues were fixed in 4% paraformaldehyde for 24 h, dehydrated, hyalinized, paraffin-embedded, and sectioned at 5 μm. Sections were stained with hematoxylin and eosin (H&E) (Sigma-Aldrich, St. Louis, MO, USA) and examined with an ordinary fluorescence microscope (Osbalin, Tokyo, Japan, magnification: 20×, 40×) to evaluate the tissue structure.

### 2.6. Intestinal Flora Analysis

Cecal contents of 6 randomly selected samples were taken. DNA was extracted from fecal samples using the QIAamp DNA Stool Mini Kit (Qiagen, Düsseldorf, Germany), and the integrity and purity of isolated DNA was assessed using a NanoDrop 2000 (Thermo Fisher Scientific, Waltham, MA, USA) and agarose gel electrophoresis. Subsequently, the 16 S rRNA V3-V4 region was amplified by PCR targeting the 16 S rDNA V3-V4 region using primers 515 F(5″-GTGCCAGCMGCCGCGGTAA-3″) 806R(5″-GGACTACHVGGGTWTCTAAT-3″). The PCR products were subsequently purified. The PCR products were then purified and quantified. Libraries were prepared using the TruSeqTM DNA Sample Preparation Kit and sequenced on the HiSeq PE250 platform (Illumina, San Diego, CA, USA). Quality assurance was performed using Fastp software (version 0.19.6, login date: 2023-11-05). Sequence assembly was performed using FLASH software (version 1.2.7) and noise reduction was achieved by the DADA2 (v2.8.1) algorithm to provide representative sequence and abundance data for amplicon sequence variants (ASVs). Species were annotated and subsequently analyzed for community diversity, composition, and species differentiation based on ASV data (phyloseq, v1.46.0).

### 2.7. Short-Chain Fatty Acid Analysis

The contents of the cecum were selected from the same numbered cecum as for 16S rDNA sequencing. The intestinal contents are extracted in acidified water, then the digested sample is thawed and suspended in 1 mL of water containing 25% metaphosphoric acid and homogenized with a vortex for approximately 2 min. The suspension is incubated at 4 °C for 30 min and centrifuged at 12,000× *g* for 10 min at 4 °C to obtain a supernatant. The internal standard 2-ethylbutyric acid solution was spiked into the supernatant at a final concentration of 1 mM prior to analysis. The sample was injected into a gas chromatography system (GC-2014, Shimadzu Corporation, Kyoto, Japan) to analyze the results. The column oven program was optimized as follows: an initial temperature of 110 °C for 30 s, heating at a rate of 10 °C/min to 120 °C, 120 °C for 4 min, and a gradual increase to 150 °C over 3 min. The concentration of each SCFA was determined from standard curves obtained from six different concentrations.

### 2.8. Statistical Analysis

Data are expressed as mean ± SEM. Continuous variables were assessed for normality using Shapiro–Wilk tests. Normally distributed data were analyzed by one-way analysis of variance (ANOVA) with homogeneity of variance confirmed by Levene’s test, while non-normally distributed data (including gut microbiota composition) were evaluated using the Kruskal–Wallis nonparametric test followed by Mann–Whitney *U* tests. Significant associations were determined by Pearson’s correlation analysis. Significant associations were defined as |ρ| > 0.5 with FDR-adjusted *p* < 0.05. All analyses were performed using SPSS 22.0 (IBM Corp., Armonk, NY, USA) and GraphPad Prism 10.1.2.

## 3. Results

### 3.1. Effects of MTE and MLE Interventions on Fat Accumulation and Serum Metabolic Parameters in Obese Mice

**Figure 2:**
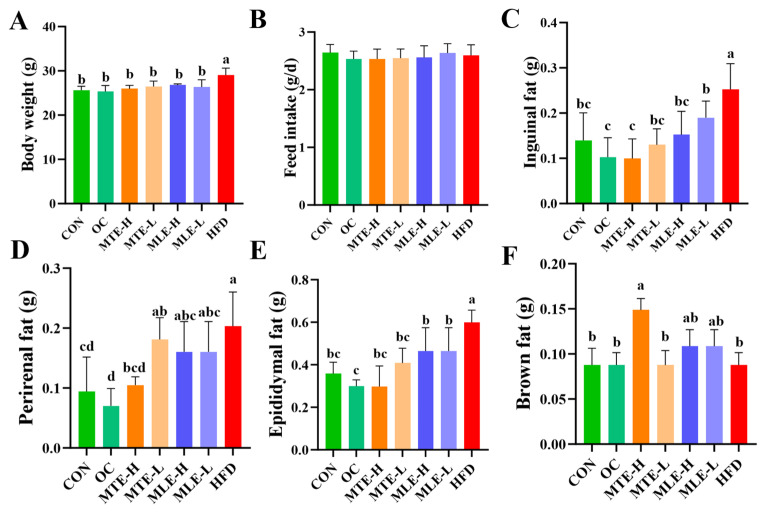
Effects of MTE and MLE interventions on fat accumulation in obese mice (*n* = 8/group). (**A**) Final body weight; (**B**) Feed intake; (**C**) Weights of inguinal fat; (**D**) Perirenal fat; (**E**) Epididymal fat; (**F**) Brown fat. Data were expressed as mean ± SD and analyzed by ordinary one-way ANOVA compared to the HFD group (model group). Different letters represent different significances (*n* = 8). Different letters above bars denote significant differences (*p* < 0.05, one-way ANOVA with Tukey’s post hoc test).

Compared with the CON group, mice in the HFD group had a significant increase in body weight after 15 weeks of feeding (*p* < 0.05). MTE, MLE, and OC interventions all significantly inhibited body weight gain in obese mice (*p* < 0.05), and this effect was not induced by changes in feed intake (*p* > 0.05). In terms of body fat deposition, the weights of white adipose tissue (inguinal fat, epididymal fat, and perirenal fat) in the HFD group were significantly higher than those in the CON group (*p* < 0.05). The MTE and MLE interventions significantly reduced the weights of inguinal and epididymal fat (*p* < 0.05), and among them, only the MTE-H group significantly reduced the accumulation of perirenal fat (*p* < 0.05). Notably, the MTE-H group also exhibited a unique metabolic improvement with a significant increase in brown adipose tissue weight compared to the HFD group (*p* < 0.05, Figure 2).

**Figure 3:**
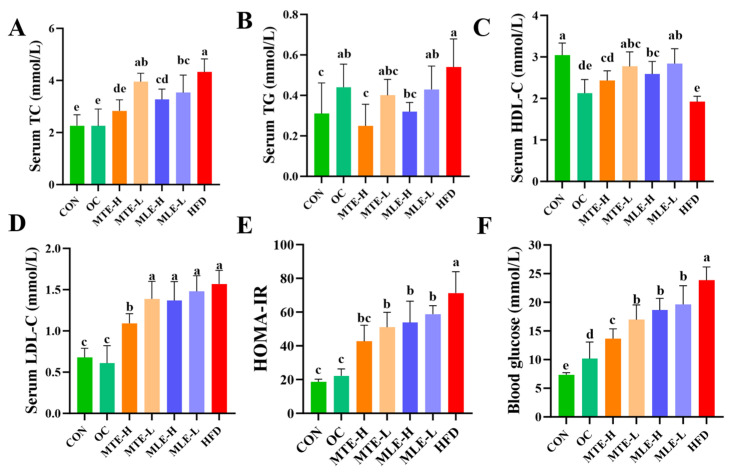
Effects of MTE and MLE on blood glucose and lipid levels in obese mice (*n* = 6/group). Serum (**A**) Cholesterol; (**B**) Triglycerides; (**C**) HDL Cholesterol; (**D**) LDL Cholesterol; (**E**) Insulin Resistance index; (**F**) Fasting blood glucose level. Data were expressed as mean ± SD and analyzed by ordinary one-way ANOVA compared to the HFD group (model group). Different letters represent different significances (*n* = 8). Different letters above bars denote significant differences (*p* < 0.05, one-way ANOVA with Tukey’s post hoc test).

**Figure 4:**
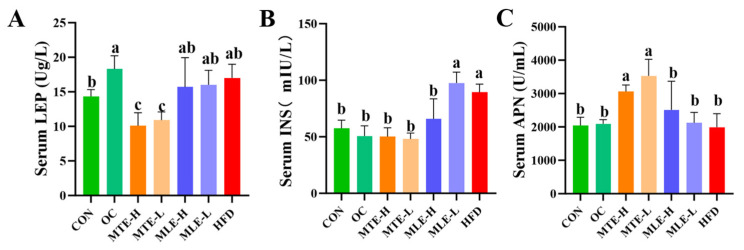
Effect of MTE and MLE on serum hormone levels in obese mice (*n* = 6/group). Serum levels of (**A**) Leptin; (**B**) Insulin; (**C**) Adiponectin. Data were expressed as mean ± SD and analyzed by ordinary one-way ANOVA compared to the HFD group (model group). Different letters represent different significances (*n* = 8). Different letters above bars denote significant differences (*p* < 0.05, one-way ANOVA with Tukey’s post hoc test).

Serum total cholesterol (TC), triglyceride (TG), low-density lipoprotein cholesterol (LDL-C), insulin resistance index (HOMA-IR), and fasting blood glucose levels were significantly elevated in the obese mice (*p* < 0.05), while the high-density lipoprotein cholesterol (HDL-C) level was significantly reduced (*p* < 0.05). MTE and MLE interventions significantly improved these metabolic abnormalities, with the MTE-H group having the most significant effect (*p* < 0.05), effectively reducing TC, TG, LDL-C, HOMA-IR, and fasting blood glucose levels, and elevating HDL-C levels. Notably, the positive control OC group showed a superior modulating effect to the mulberry extract in all metabolic indexes except TG (*p* < 0.05, Figure 3). INS levels were significantly higher in the HFD group compared with the CON group (*p* < 0.01). After MTE and MLE interventions, INS levels were significantly reduced in all treatment groups except the MLE-L group (*p* < 0.01). In terms of LEP regulation, all doses of MTE treatment significantly reduced LEP levels (*p* < 0.01), which was better than that of the positive control OC group, while MLE treatment had no significant effect on LEP levels (*p* > 0.05). In terms of APN regulation, MTE significantly increased the APN level, while MLE brought the APN level close to that of the OC group, but the difference was not significant compared with that of the HFD group (*p* > 0.05, Figure 4).

### 3.2. Effects of MTE and MLE Interventions on Hepatic Metabolic Parameters in Obese Mice

**Figure 5:**
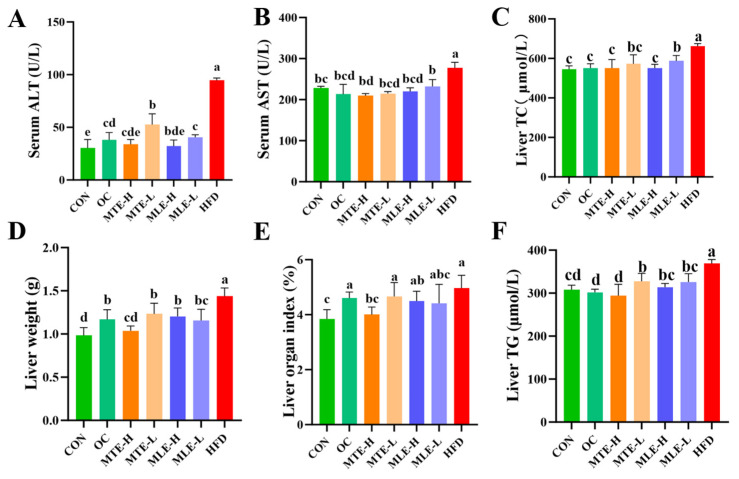
Effects of MTE and MLE on liver function and metabolic indexes of obese mice (*n* = 6/group). (**A**) Serum alanine aminotransferase; (**B**) Serum glutamate aminotransferase; (**C**) Hepatic cholesterol; (**D**) Hepatic weight; (**E**) Hepatic organ index; (**F**) Hepatic triglyceride levels. Data were expressed as mean ± SD and analyzed by ordinary one-way ANOVA compared to the HFD group (model group). Different letters represent different significances (*n* = 8). Different letters above bars denote significant differences (*p* < 0.05, one-way ANOVA with Tukey’s post hoc test).

Compared to the CON group, the ALT and AST levels in the serum of the HFD group mice were significantly higher (*p* < 0.05), indicating that the obese mice had significant liver damage. At the same time, the weight of the liver, the liver index, and the liver TG and TC content were all significantly increased (*p* < 0.05). After MTE and MLE intervention, all treatment groups significantly improved the above abnormal indicators (*p* < 0.05), with the MTE-H group showing the best hepatoprotective effects: improving liver function indicators (AST and ALT) slightly better than the OC group and significantly improving liver fat accumulation (liver weight, liver index, and TG and TC content) compared to the OC group (*p* < 0.05, Figure 5).

**Figure 6:**
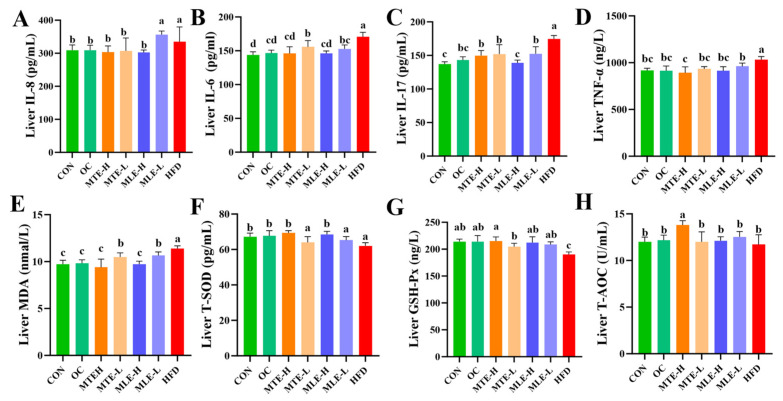
Effects of MTE and MLE on liver inflammatory factors and oxidative stress indices in obese mice (*n* = 6/group). (**A**) Interleukin-8 (IL-8); (**B**) Interleukin-6 (IL-6); (**C**) Interleukin-17 (IL-17); (**D**) Tumor Necrosis Factor-α (TNF-α); (**E**) Malondialdehyde (MDA); (**F**) Total Superoxide Dismutase (T-SOD); (**G**) Glutathione Peroxidase (GSH-Px); (**H**) Total antioxidant capacity (T-AOC). Data were expressed as mean ± SD and analyzed by ordinary one-way ANOVA compared to the HFD group (model group). Different letters represent different significances (*n* = 8). Different letters above bars denote significant differences (*p* < 0.05, one-way ANOVA with Tukey’s post hoc test).

Compared to the CON group, the HFD group had significantly higher levels of pro-inflammatory factors (IL-8, IL-6, IL-17, and TNF-α) (*p* < 0.05), as well as increased levels of the oxidative stress marker MDA (*p* < 0.05), while the antioxidant indicators (T-SOD, GSH-Px, and T-AOC) were significantly lower (*p* < 0.05). MTE and MLE interventions significantly improved these abnormal indicators (*p* < 0.05, Figure 6).

MTE-H exhibited the best improvement effects, with a comparable inhibitory effect on inflammatory factors to the OC group. Notably, MTE-H significantly increased T-SOD, GSH-Px activity, and T-AOC levels (*p* < 0.05). Notably, MTE-H significantly increased T-AOC levels more than other treatment groups (*p* < 0.05, Figure 6).

### 3.3. Pathologic Examination of Liver Tissue Sections

**Figure 7:**
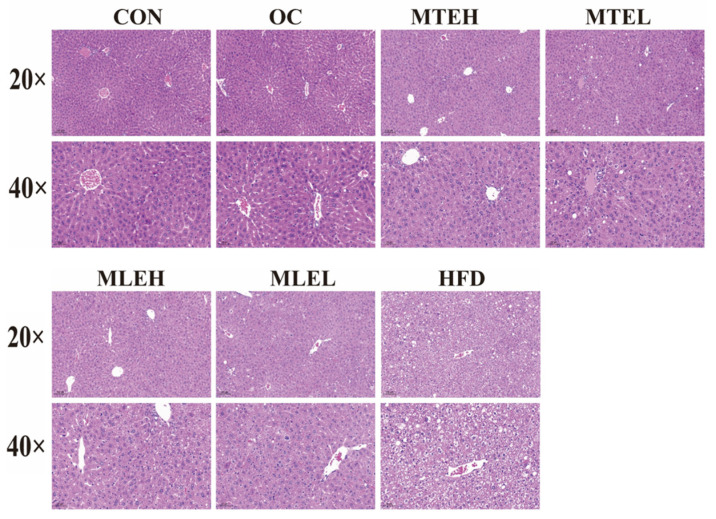
Effect of MTE and MLE interventions on liver lipid deposition profile. Representative images of liver morphology, H&E staining of liver tissue in CON, MTEL, MTEH, MLEH, MLEL, and HFD groups (original magnification ×20, original magnification ×40).

The histomorphology of HE-stained liver sections at different magnifications (20× and 40×) is shown below. The livers of mice in the HFD group showed a significant increase in adipocyte number and cell volume, as well as the extrusion of cell nuclei to the margins. There was also an increase in the number of hepatocyte lipid droplet vesicles, suggesting obvious steatosis or fat infiltration in obese mice. In contrast, liver tissues from the CON group showed normal cellular arrangement and morphology with uniformly stained nuclei and clear cytoplasm; no obvious pathological changes were observed. In the OC group, the number of hepatocyte lipid droplet vesicles decreased, the cells became more tightly arranged, and the nuclei became darker, suggesting a potential slight inflammatory response or cell proliferation. Additionally, the number of fat cells decreased in the MTE-H, MTE-L, MLE-H, and MLE-L groups. The liver’s transverse structure was clear, and the nuclei were located at the edge of the fibers. No obvious degeneration or necrosis was observed (Figure 7).

### 3.4. Effects of MTE and MLE Interventions on Intestinal Short-Chain Fatty Acid Metabolic Profiles in Obese Mice

**Figure 8:**
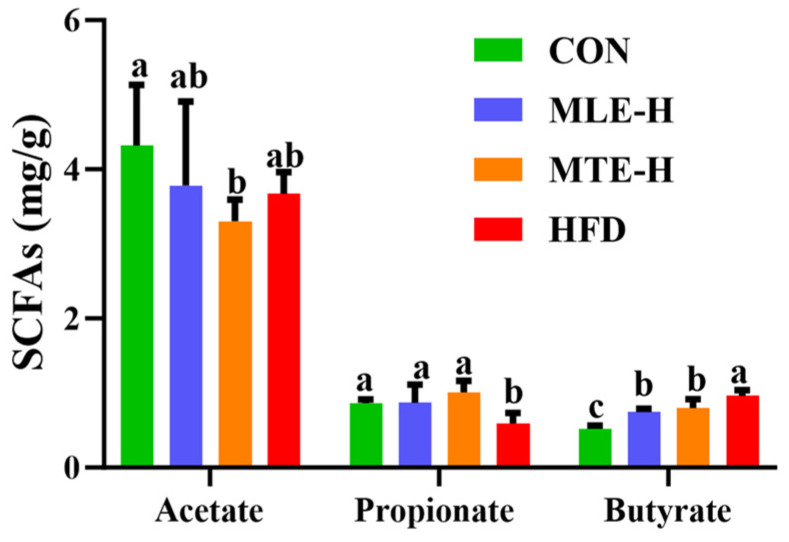
Effect of MTE and MLE interventions on liver lipid deposition profiles (*n* = 6/group). Data are presented as the mean ± SD (*n* = 6). Different letters above bars denote significant differences (*p* < 0.05, one-way ANOVA with Tukey’s post hoc test).

Compared with the CON group, the propionic acid content in the cecum was significantly decreased, while the butyric acid content was significantly increased (*p* < 0.05) in the HFD group. The acetic acid content was unaffected (*p* > 0.05). The MTE-H and MLE-H interventions significantly reversed the changes in cecum propionic and butyric acid content in mice fed a high-fat diet compared with the HFD group, while there was no significant effect on acetic acid content (Figure 8).

### 3.5. Effects of MTE and MLE Interventions on the Diversity of Intestinal Flora in Obese Mice

**Figure 9:**
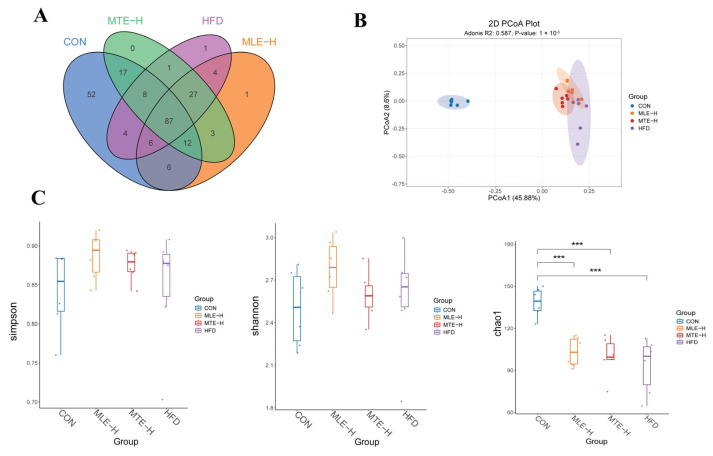
Effect of MTE and MLE on the diversity of intestinal flora in obese mice (*n* = 6/group). (**A**) Venn diagram; (**B**) PCoA analysis; (**C**) Alpha-diversity analysis (Chao1, Shannon and Simpson indices). ***, *p* < 0.001 vs. control group (one-way ANOVA with Tukey’s multiple comparisons test).

The ASVs were obtained using the noise reduction method and analyzed for common and unique ASVs among the different treatment groups. The results showed that there were 87 shared ASVs and 52, 17, 1, and 1 unique ASV among the CON, MTE-H, MLE-H, and HFD groups, respectively (Figure 9A). Compared with blank control mice, obese mice had a significantly lower Chao1 index (*p* < 0.05). However, there was no significant difference in the Shannon and Simpson indices compared with the CON group (*p* > 0.05) (Figure 9C). Principal coordinate analysis (PCoA) showed a significant difference in intestinal flora composition between the HFD group and the CON group (*p* < 0.05). Meanwhile, the intestinal flora structure of the MTE-H and MLE-H intervention groups tended to regress toward the CON group (Figure 9B).

### 3.6. Effects of MTE and MLE on the Composition of Intestinal Flora in Obese Mice

**Figure 10:**
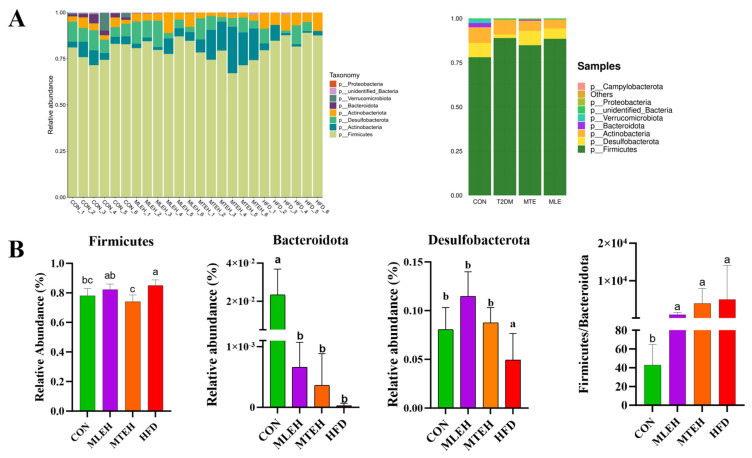
Effect of MTE and MLE on the composition of intestinal flora at the portal level in obese mice (*n* = 6/group). (**A**) Relative abundance of TOP 10 bacteria at the gate level for each sample and treatment group; (**B**) relative abundance and Firmicutes/Bacteroidota (F/B) ratio of bacteria differing at the gate level; data are presented as mean ± SD deviation, and different letters indicate statistical differences between the two data sets (*p* < 0.05).

At the phylum level, results showed that a high-fat diet significantly increased the relative abundance of *Firmicutes*, *Actinobacteriota*, and *Desulfobacterota* and decreased the relative abundance of *Bacteroidota* compared to the control group (*p* < 0.05). Compared to the high-fat diet group, the MTEH intervention significantly reduced the relative abundance of *Actinobacteriota* and *Firmicutes* (*p* < 0.05), while the MLEH intervention did not have a significant effect (*p* > 0.05). The *Firmicutes*-to-*Bacteroidetes* ratio was significantly increased in the HFD, MLEH, and MTEH groups compared to the CON group. After the MLEH and MTEH interventions, the proportion of *Firmicutes/Bacteroidetes* decreased compared with the HFD group. The MTEH intervention was more effective, though the difference was not significant (*p* > 0.05, Figure 10).

**Figure 11:**
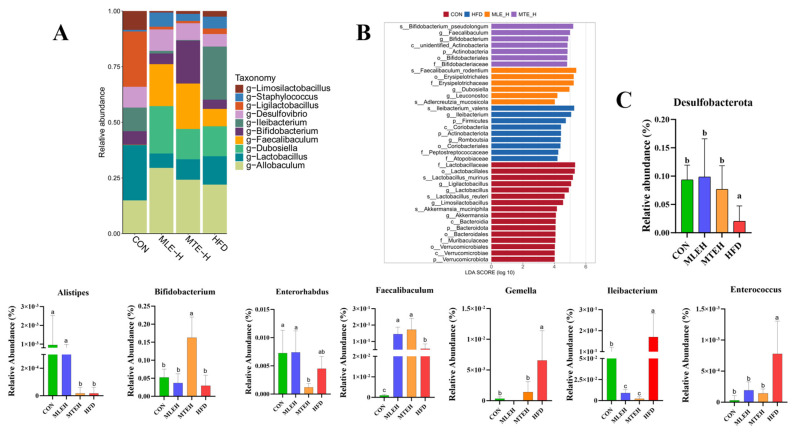
Effect of MTE and MLE on gut flora composition at the genus level in obese mice (*n* = 6/group). (**A**) Relative abundance of genus-level TOP 10 bacteria; (**B**) linear discriminant effect size analysis (LefSe) to identify representative bacterial taxa at different taxonomic levels for each treatment group; (**C**) relative abundance of genus-level differential bacteria; data are presented as mean ± SD deviation, and different letters indicate statistical differences between the two data sets (*p* < 0.05).

At the genus level, both the MLEH and MTEH interventions significantly reduced the relative abundance of *Leibia*, *Gemella*, and *Enterococcus* (*p* < 0.05), while promoting the relative abundance of *Desulfobacterota* and *Faecalibaculum*. *Faecalibaculum* enrichment was also significant (*p* < 0.05). Notably, the MLEH intervention increased the abundance of *Alistipes* and *Enterorhabdus*, whereas the MTEH intervention suppressed the abundance of these two genera and boosted the abundance of *Bifidobacterium* (Figure 11).

Using LEfSe analysis, we identified microbial markers with an LDA score greater than 2 that differed significantly between groups. This revealed the specific regulatory effects of different interventions on cecum flora structure. The HFD group was significantly enriched in *g-Ileibacterium*, the MTEH group was enriched in *g-Faecalibaculum* and *g-Bifidobacterium*, and the MLEH group was enriched in *g-Dubosiella* and *g-Leuconostoc* (Figure 11B).

### 3.7. Correlation Analysis of Gut Microorganisms and Short-Chain Fatty Acid Profiles

**Figure 12:**
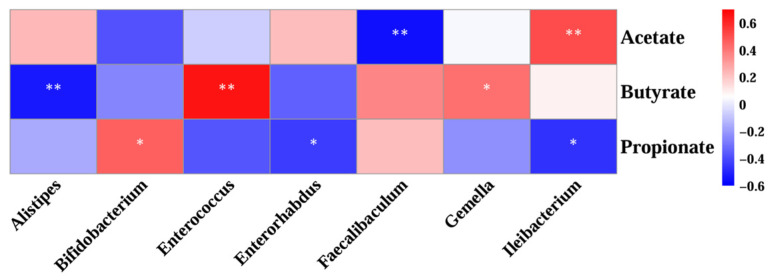
Correlation analysis between genus-level gut microbes and short-chain fatty acids. Each grid represents the Spearman correlation coefficient between rows and columns. A red color indicates positive correlation and a blue color indicates negative correlation. Significance: * 0.01 < *p* < 0.05; ** *p* < 0.01.

Spearman’s correlation was used to assess the relationship between SCFAs and gut microbes at the genus level (Figure 12). *Faecalibaculum*, a genus that was significantly enriched after both MLEH and MTEH interventions, was negatively correlated with acetate (r = −0.5260, *p* < 0.01; r = −0.435, *p* < 0.05). *Alistipes* and *Enterorhabdus*, specifically enriched by the MLEH intervention, showed a strong correlation (r = −0.560, *p* < 0.01) with butyrate and propionate metabolism, respectively. Strong correlations were observed for *Bifidobacterium* spp., which were specifically enriched after MTEH treatment, with propionate metabolism (r = 0.460, *p* < 0.05).

## 4. Discussion

Obesity has been shown to lead to dysbiosis of the intestinal flora, steatosis, liver inflammation, and impaired glucose homeostasis [39]. Orlistat (OC) is an effective weight loss medication that controls body weight by inhibiting lipase in the gastrointestinal tract and decreasing fat absorption, thereby reducing caloric intake [15]. However, long-term use of orlistat has been shown to cause gastrointestinal distress, impede the absorption of fat-soluble vitamins, and lead to cholelithiasis, kidney stones, and liver damage [40]. Our findings suggest that different doses of MTE and MLE have similar obesity-resistant effects with OC. Different doses of MTE and MLE reduced body weight, liver weight, and fat mass in obese mice, similar to previous studies [41]. However, the MTEH-treated group significantly increased in brown fat content, which was not present in the OC treatment. Brown fat is a promising way to address nutritional imbalances in the human body because it can enhance glucose metabolism, reduce adiposity, and improve insulin sensitivity [42]. Brown fat can be converted from white fat when properly stimulated [43]. This change may occur due to the activation of adipose tissue thermogenesis-related signaling pathways, such as the cAMP-PKA-MAPK pathway, as well as regulatory hormones and receptors that promote the browning of white adipose tissue through natural components (e.g., quercetin and rutin) found in MTE [44,45,46]. Therefore, MTE exhibited a more efficient capacity for fat conversion and energy expenditure compared with the OC treatment.

This study found that MTE and MLE intake significantly suppressed excessive fat accumulation in the adipose tissue and liver of mice fed a high-fat diet. High-fat diets typically lead to elevated levels of INS and LEP. LEP is secreted by adipocytes, while INS is secreted by pancreatic islet cells. LEP levels are proportional to body fat content [47]. LEP levels within the normal range promote weight loss. Supplementation with MTE and MLE inhibited the elevation of LEP and INS caused by a high-fat diet and reduced insulin resistance, leptin resistance, and blood glucose levels. Different doses of MTE and MLE supplementation increased serum APN levels; the MTE group had the best effect, superior to the OC group. These results further revealed the beneficial effects of mulberry extract on obese mice’s metabolism.

Obesity is characterized by hepatic steatosis and inflammation. Inflammation in adipose tissue can trigger obesity-related cardiovascular diseases and type 2 diabetes. The liver is directly connected to the gastrointestinal tract via the portal and biliary veins, so it is frequently exposed to products derived from the gut microbiota. Under certain conditions, gut symbionts and their byproducts can travel from the intestinal lumen to the liver, where they can influence hepatic immune responses. Therefore, the gut microbiota play a crucial role in liver health [48,49,50,51]. Transaminases (AST and ALT), which are enzymes found in the liver, are indicators of liver dysfunction and injury [52]. Our study found that MTE and MLE intake achieved a similar attenuation of liver injury to what OC did by reducing serum AST and ALT activities. This demonstrates the protective effect of MTE and MLE on the livers of obese mice. This study also showed that MTE and MLE interventions prevented oxidative damage by normalizing T-AOC, T-SOD, GSH-Px, and MDA levels. To investigate whether MTE and MLE could reduce inflammation, we examined changes in hepatic inflammatory factors. Macrophages are major inflammatory and immune effector cells that play a crucial role in producing pro-inflammatory cytokines (e.g., TNF-α, IL-6, IL-17, and IL-8) [53,54,55]. In this study, TNF-α, IL-6, IL-17, and IL-8 levels were significantly lower in the livers of high-fat diet (HFD) mice after MTE and MLE interventions. These results suggest that MTE and MLE interventions positively affect the attenuation of oxidative stress and inflammation in the livers of obese mice.

The present study examined the effects of a high-fat diet on intestinal acid content. Propionic acid content decreased, butyric acid content increased, and there was no significant effect on acetic acid content. Propionic acid is one of the SCFAs produced primarily in the colon. It enters the liver and reduces lipid content by altering hepatic metabolic processes [56]. *Bacteroidota* have been shown to form propionic acid from dietary carbohydrates via the succinate pathway [57]. In the present study, *Bacteroidota* abundance decreased significantly after high-fat dietary treatments. Propionic acid content was higher in obese mice after MTEH and MLEH interventions. The fluctuation of propionic acid content in this experiment may be related to changes in *Bacteroidota* abundance. In this study, the content of butyric acid in the ceca of obese mice decreased after MTEH and MLEH treatments. The abundance of *Firmicutes* significantly decreased in the MTEH group. A high-fat diet has been shown to significantly alter the composition of intestinal flora, increasing the abundance of *Firmicutes* and contributing to energy absorption by increasing the production of short-chain fatty acids [58,59]. Additionally, acetic acid can be produced through various pathways, and the production and diversity of acetic acid sources may explain the absence of significant changes in acetic acid levels in the intestines of mice fed a high-fat diet [60].

In obese mice, an increase in *Lactobacillus* spp. may lead to altered intestinal pH and disturbed bile acid metabolism, which affects the structure and function of intestinal flora and influences the host’s metabolic status [61]. This study found that the relative abundance of *Allobaculum* and *Lactobacillus* increased significantly in obese mice after receiving different doses of MTE and MLE. This increase may be related to the mechanism by which mulberry extracts inhibit lipase activity and improve the intestinal barrier. This, in turn, affects lipid metabolism and regulates lipid absorption and storage. An increase in *Faecalibaculum*, a group of beneficial, butyrate-producing intestinal bacteria, has been associated with significant reductions in markers of intestinal inflammation and oxidative stress in obese mice [62]. However, an increase in *Gemella* may inhibit the growth of *Faecalibaculum*, thereby affecting the overall function of the intestinal flora. Additionally, an increase in *Ileibacterium* may negatively affect *Faecalibaculum* abundance [63]. The relative abundance of *Gemella* and *Ileibacterium*, as well as *Faecalibaculum* abundance, was significantly reduced in obese mice after MTEH and MLEH treatments, which may be related to the influence of *Gemella* and *Ileibacterium* on *Faecalibaculum* abundance. This may be due to the reduced negative influence of *Gemella* and *Ileibacterium* on *Faecalibaculum* growth. Therefore, *Faecalibaculum* may ameliorate obesity by regulating intestinal flora structure, promoting short-chain fatty acid production, enhancing intestinal barrier function, and improving metabolic health.

Several studies have shown that high-fat dietary treatments significantly increase the relative abundance of *Desulfovibrio*, a bacterium that produces lipopolysaccharides which damage the intestinal barrier and exacerbate inflammation [64,65]. While these findings describe a positive association between *Desulfovibrio* and disease, some studies have reported the opposite [66,67]. Supplementing Astragalus polysaccharide in mice with non-alcoholic fatty liver disease (NAFLD) specifically increased the abundance of Desulfovibrio, increased acetic acid-dominant SCFAs, and suppressed the expression of the key protein for hepatic lipid synthesis, fatty acid synthase (*FASN*)/*CD36*. In vitro assays demonstrated that *Desulfovibrio* can be cultured anaerobically. In vitro experiments showed that *Desulfovibrio* can efficiently produce acetic acid in an anaerobic environment. This suggests that *Desulfovibrio* can effectively reduce hepatic steatosis by producing acetic acid and regulating hepatic lipid metabolism in mice [68]. These findings challenge the traditional perception that sulfate-reducing bacteria are solely detrimental. In the present study, *Desulfovibrio* abundance significantly increased in obese and T2DM mice after MTE and MLE interventions. This suggests that changes in *Desulfovibrio* may improve glucolipid metabolism by affecting H_2_S metabolism and participating in inflammatory regulation. *Bifidobacterium* play an important role in fighting obesity through mechanisms such as regulating intestinal microecology, improving metabolic health, and reducing inflammation and oxidative stress [69]. MTE treatment was shown to significantly increase the relative abundance of *Bifidobacterium* in the intestines of obese mice, which may be inextricably linked to MTE’s anti-inflammatory and antioxidant effects.

MTE may have anti-obesity properties. It increases the abundance of *Bifidobacterium* in the intestinal tract of obese mice. This increase occurs through various mechanisms. These mechanisms include regulating the structure of intestinal flora, promoting the production of short-chain fatty acids, enhancing intestinal barrier function, improving metabolic health, and modulating the immune response. Mulberry twig and leaf extracts (MTEs and MLEs) are promising natural alternatives to antibiotic feed additives because they coordinate the modulation of inflammatory pathways, intestinal microbiota composition, and microbial metabolite production. Unlike single-target interventions, MTE and MLE exert multifaceted therapeutic effects by modulating multiple obesity-related metabolic pathways simultaneously, offering superior potential for metabolic regulation in livestock production. In this study, the regulation of metabolic health by MTE and MLE through gut microbiota-induced SCFA changes was based solely on Spearman’s correlation, lacking causal validation, and thus has certain limitations. Future studies should conduct fecal microbiota transplantation trials and use germ-free animal models for mechanistic validation.

## 5. Conclusions

Mulberry extract intervention inhibited the increases in body weight, inguinal lipids, epididymal lipid weight, blood lipids, HOMA-IR, blood glucose, hormone levels, liver weight, hepatic organ index, and hepatic inflammatory factor levels induced by a high-fat diet. It also inhibited the reduction in serum AST, ALT, hepatic lipid deposition, and hepatic MDA levels. The mulberry extract also inhibited reductions in hepatic T-SOD, GSH-Px, and T-AOC levels and serum APN and HDL-C levels. Specifically, the MTE-H group reduced perirenal lipid weight and significantly increased brown fat weight. Mulberry extract intervention significantly increased the propionic acid level in the cecum and decreased the butyric acid level in obese mice. MTEH intervention significantly decreased the perirenal lipid weight in obese mice.

The MLEH intervention significantly decreased the relative abundance of the phyla *Actinobacteriota* and *Firmicutes* in obese mice. The MLEH and MTEH interventions significantly decreased the relative abundance of the genera *Lachnospiraceae*, *Gemella*, and *Enterobacteriaceae* in obese mice.

**Figure 13:**
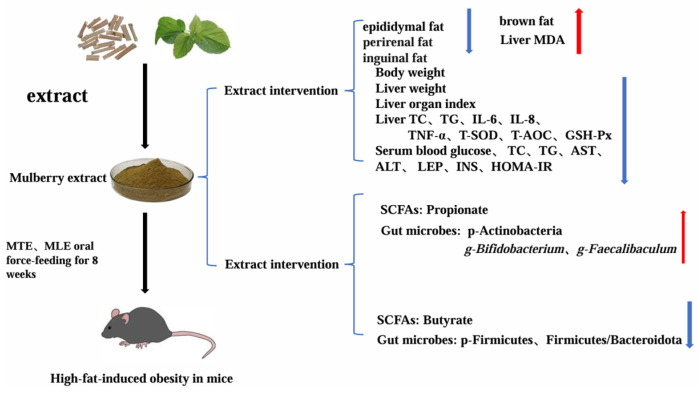
Mechanisms of MTE and MLE in Attenuating Lipid Deposition in Obese Mice. MTE, mulberry twig extract; MLE, mulberry leaf extract;red arrows indicate an increase, blue arrows indicate a decrease.

It promoted the enrichment of *Desulfovibrio* and *Faecalibaculum* while decreasing the abundance of *Gemella* and *Enterococcus*. The MLEH intervention specifically increased the abundance of *Alistipes* and *Enterorhabdus*. The MTEH intervention showed the opposite trend, significantly suppressing the abundance of these two genera and significantly increasing the abundance of *Bifidobacterium* (Figure 13).

## Data Availability

The data presented in this study are available on request from the corresponding author. The data are not publicly available due to their integral role in an ongoing research program requiring phased disclosure.
